# *Curcuma raktakanda* Induces Apoptosis and Suppresses Migration in Cancer Cells: Role of Reactive Oxygen Species

**DOI:** 10.3390/biom9040159

**Published:** 2019-04-23

**Authors:** Shruti Mishra, Sumit Singh Verma, Vipin Rai, Nikee Awasthee, Jayadev S. Arya, Kaustabh K. Maiti, Subash C. Gupta

**Affiliations:** 1 Laboratory for Translational Cancer Research, Department of Biochemistry, Institute of Science, Banaras Hindu University, Varanasi-221 005, India; shruti25mishra87@gmail.com (S.M.); sumit.mhg.bhu14@gmail.com (S.S.V.); vipinrai28@gmail.com (V.R.); itsnikee@gmail.com (N.A.); 2 CSIR-National Institute for Interdisciplinary Science and Technology, Chemical Science and Technology Division, Organic Chemistry Section, Trivandrum-695019, India; aryaas2010@gmail.com (J.S.A.); kkmaiti29@gmail.com (K.K.M.)

**Keywords:** cancer, curcuma, glioblastoma, inflammation, reactive oxygen species

## Abstract

Although over 100 species of Curcuma are reported, only *Curcuma longa* is extensively studied. *Curcuma raktakanda*, a poorly studied species, is most commonly distributed in the Kerala state of India. For the first time, we examined the efficacy of different fractions (acetone, hexane, and ethyl acetate) of *C. raktakanda* against glioma, cervical, and breast cancer cell lines. As determined by mitochondrial reductase activity assay, the viability of cancer cells was decreased in a concentration-dependent manner by the three fractions. The half maximal inhibitory concentration (IC-50) values after the treatment of C-6 glioma cells for 48 h was found to be 32.97 µg/mL (acetone extract), 40.63 µg/mL (hexane extract), and 51.65 µg/mL (ethyl acetate extract). Of the three fractions, the acetone fraction was more effective. The long-term colony formation of cancer cells was significantly suppressed by the acetone fraction. Analyses using DAPI (4′,6-diamidino-2-phenylindole) staining, AO/PI (acridine orange/propidium iodide) staining, DNA laddering, and sub-G1 population revealed that the acetone extract induced apoptosis in glioma cells. The extract induced reactive oxygen species generation and suppressed the expression of cell survival proteins. The migration of cancer cells was also suppressed by the acetone extract. The gas chromatography-mass spectrometry (GC-MS) analysis indicated that tetracontane, dotriacontane, hexatriacontane, pentacosane, hexacosane, and eicosane are the major components in the acetone extract. Collectively, the extract from *C. raktakanda* exhibited anti-carcinogenic activities in cancer cells. We are exploring whether the phytoconstituents, individually, or collectively contribute to the anti-cancer activities of *C. raktakanda*.

## 1. Introduction

Glioblastoma multiforme (GBM, glioblastoma or grade IV glioma) is the most aggressive, invasive, and most common tumor of the central nervous system [1]. These tumors arise from astrocytes of the human brain and show high resistance to currently available therapy. Despite the significant advances in surgery, radiation therapy, and chemotherapy, the prognosis remains poor for glioblastoma with a median survival of 15.4 months after diagnosis [2]. Because of the poor penetration of the blood brain barrier and development of drug resistance, the chemotherapy options for glioblastoma are limited [3,4]. Although some drugs such as temozolomide have been approved, glioblastoma remains highly incurable [5]. The implication of these reports necessitates the development of novel therapy that can be used either as a single agent or as an adjuvant for glioblastoma therapy. 

The natural products have been used for the treatment of several illnesses including cancer since ancient times. Globally, 80% of the human population are estimated to rely on plant-derived medicines for their health care needs [6]. Further, 49% of the small molecules approved for cancer therapy between 1940 and 2014 had originated from nature [6,7]. Plant derived medicines have played a pivotal role in the healthcare of both ancient and modern society [8,9,10,11]. Paclitaxel (taxol), is one of the success stories for the use of agents derived from natural sources in cancer therapy. Originally isolated from the bark of the Pacific yew tree in 1971, this antimitotic agent has been reported to be effective against several cancer types including breast, ovarian, pancreatic, and non-small cell lung cancer [12,13,14]. Because of multitargeting, low cost, safety, and ease of availability, interest in natural products for cancer therapy continues to grow. 

The genus Curcuma (family Zingiberaceae) is one of the widely studied medicinal plants, the extract and constituents of which have demonstrated anti-cancer activities. Although as many as 133 species have been identified from this genus, only *Curcuma longa*, the golden spice turmeric, is being extensively studied. Curcumin, a minor but the most active component of turmeric, is reported to exhibit activities both in animal models and in humans [15,16,17,18]. Recent studies suggest that some species of curcuma are as active or even more active than *C. longa*. For example, the rhizomes of *C. phaeocaulis* is reported to exhibit better anti-inflammatory activity compared to *C. longa* [19]. 

However, studies on the anti-cancer activities of species other than *C. longa* are very few. *Curcuma raktakanda* is one such poorly studied species, which is widely distributed in the Kerala state of India [20]. One study examined the effects of *C. raktakanda* extracts (leaves and tuber) on the early fourth instar larvae of four mosquito species (*Aedes aegypti, Anopheles stephensi, Culex quinquefasciatus*, and *Culex sitiens*). The petroleum ether extract (leaves and tuber) induced toxicity in all the tested mosquito species [20]. However, the active constituent from the extract responsible for the larvicidal activities was not examined. Whether the extract and the constituents from *C. raktakanda* exhibit anti-cancer activity has not been reported previously. However, non-cancer drugs such as antibiotics, antiepileptics, anesthetics, and cardioprotectives have been successfully explored for anti-cancer activities [21].

Because glioblastoma, like other cancer types, is a multigenic disease, the current paradigm for the therapy is either to combine multiple mono-targeted agents or to design a molecule that can target multiple pathways. Since, the extract is a mixture of several components, we sought to investigate the efficacy of *C. raktakanda* extract against glioblastoma. Additionally, we examined the efficacy of the extract against breast cancer and cervical cancer. The results to be discussed suggest that the *C. raktakanda* extract suppresses the viability of wide variety of cancer cells. Furthermore, the extract induces apoptosis and suppresses the migration of cancer cells.

## 2. Material and Methods

### 2.1. Plant Extract 

The three extracts (hexane, ethyl acetate, and acetone) were obtained from the rhizome of *C. raktakanda*. The extraction was done in collaboration with Dr. Maiti from NIIST (CSIR) Trivandrum, India. *Curcuma raktakanda* rhizomes were collected from the Jawaharlal Nehru Tropical Botanical Garden and Research Institute (JNTBGRI) and the Medicinal Plant Garden Thiruvananthapuram in February 2014. In brief, the rhizomes were thoroughly cleaned, dried at 40 °C for three days, powdered, and approximately 500 g was weighed out for further processing. The extraction was carried out from the powdered material in a successive manner using hexane (1.5 L), ethyl acetate (1.5 L), and acetone (1.5 L). The extraction was performed three times with each solvent at room temperature. Finally, Buchi rotary evaporator (Mumbai, Maharashtra, India)was used to concentrate the extract under reduced pressure. The total yield was found to be around 30g (hexane extract), 25g (ethyl acetate extract), and 25g (acetone extract). 

### 2.2. Reagents 

Dulbecco’s modified eagle medium (DMEM), Roswell Park Memorial Institute 1640 (RPMI-1640), penicillin, streptomycin, and trypsin-EDTA (ethylenediaminetetraacetic acid) were procured from Himedia (Mumbai, Maharashtra, India). Crystal violet, dimethyl sulfoxide (DMSO), and 3-[4,5-dimethylthiazol-2-yl]-2,5-diphenyl tetrazolium bromide (MTT) were obtained from SRL Diagnostics (Mumbai, Maharashtra, India). The 2′,7′-dichlorodihydrofluorescein diacetate (H2DCFDA), 4′,6-diamidino-2-phenylindole (DAPI), 5,5′,6,6′-Tetrachloro-1,1′,3,3′-tetraethyl benzimidazolyl carbocyanineiodide (JC-1), acridine orange, agarose, alexa fluor 488, ethidium bromide, fetal bovine serum (FBS), and propidium iodide were obtained from Invitrogen (Carlsbad, CA, USA). Bcl-xL antibody was obtained from Santa Cruz Biotechnology (Santa Cruz, CA, USA) while GAPDH (glyceraldehyde-3-phosphate dehydrogenase) was obtained from Abgenex (Bhubaneswar, Odisha, India). 

### 2.3. Cell Lines 

The human breast (MDA-MB-231, MCF-7), cervical (HeLa), and rat glioma (C-6) cell lines were obtained from the National Centre for Cell Science (NCCS), Pune, India. MDA-MB-231, MCF-7, and HeLa cells were cultured in high glucose DMEM, while RPMI-1640 was used for C-6 cells. The FBS (10%), penicillin (100 units/mL), and streptomycin (100 µg/mL) were used to supplement the media. 

### 2.4. Assay for Cell Viability 

The mitochondrial reductase activity was measured to determine the effect of extracts on the viability of cancer cells using MTT as a substrate [22]. The cytotoxic potential of chemotherapeutic agents was also examined using the same assay. The cells were seeded in different wells of 96 well plate (10,000/well) and treated with different concentrations of extract for 48 h. The formation of purple formazan was measured for examining the cell viability.

### 2.5. Assay for Colony Formation 

The ability of a single cell to grow into a colony was examined by clonogenic assay, which is an in vitro cell survival assay. We used a method described previously with minor modifications [23]. For this, approximately 1000 cells were seeded per well and treated with different concentrations of the acetone extract for 6 h. The cells were washed, and the colony formation was measured after 6–7 days. Finally, the colonies were stained with 0.1% crystal violet and counted manually.

### 2.6. Assay for DNA Laddering 

DNA laddering is a distinctive feature during the late stages of apoptosis. The assay was performed using a method described earlier [24]. The cells were treated with acetone extract (25–50 µg/mL) for 24 h followed by washing and lysis in a buffer containing 5 mM EDTA, 5M NaCl, 100 mM Tris-HCl (pH 8.5), and 10% sodium dodecyl sulfate (SDS). The deproteinization was then carried out with proteinase K (20 mg/mL) at 55 °C for 2 h. The deproteinized lysate was mixed with an equal volume of chloroform and centrifuged at 12,000 rpm for 15 min at 4 °C. Subsequently, an equal volume of isopropanol was added, and the DNA was pelleted at 12,000 rpm for 20 min. The DNA was then washed with 70% ethanol, air dried, dissolved in tris-EDTA buffer, and electrophoresed on 1.2% agarose gel containing ethidium bromide. Finally, the electrophoresed DNA was visualized and photographed in a gel documentation system (BioRad Gel Doc XR^+^, Hercules, CA, USA).

### 2.7. Assay for Nuclear Morphology 

To examine the effects of the extracts on the nuclear morphology, DAPI staining was performed [22]. C-6 glioma cells were treated with the acetone extract (1–50 µg/mL) for 24 h. The cells were then washed with PBS, fixed with paraformaldehyde (4%), permeabilized with methanol, and stained with DAPI. The morphology of the nucleus was examined under the fluorescence microscope and the images were captured. 

### 2.8. Assay for Cell Viability by Staining with Acridine Orange/Propidium Iodide 

In order to examine cell viability accurately, we performed AO/PI staining. C-6 cells were treated with the acetone extract (1–50 µg/mL). After 24 h, the cells were washed with PBS, stained with AO/PI, and visualized under the fluorescence microscope.

### 2.9. Assay for Sub-G1 Population 

We stained the cells with propidium iodide (PI) to examine the effects of the acetone extract on the sub-G1 population by flow cytometry [25]. The C-6 glioma cells were treated with acetone extract for 24 h. After washing with PBS, the cells were fixed with 70% chilled methanol, treated with RNaseA, and stained with PI. The FACScan was used to determine the sub-G1 population and the analysis was performed by Cell Quest software (Becton Dickinson, San Jose, CA, USA).

### 2.10. Assay for Mitochondrial Membrane Potential (ΔΨ)

To examine the effects of acetone extract on the mitochondrial membrane potential, we used a fluorochrome JC-1 as reported earlier [26]. Briefly, the control and treated cells were stained with JC-1 (10 μg/mL), washed, and imaged first under red filter and then under green filter. While the green fluorescence indicated the cells with depolarized mitochondria, red fluorescence indicated cells with intact mitochondria.

### 2.11. Assay for Protein Expression 

Whether the acetone extract affects the expression of cell survival proteins was examined by western blot analysis [27]. The whole cell lysate, from normal and treated cells, were electrophoresed on SDS-PAGE (sodium dodecyl sulfate–polyacrylamide gel electrophoresis) and transferred onto polyvinylidene difluoride (PVDF) membrane. The membrane containing the transferred proteins were first probed with the primary antibody, then with the horseradish peroxidase (HRP) conjugated secondary antibody, and finally detected using enhanced chemiluminescence (ECL) reagent. 

### 2.12. Assay for Cell Migration 

To examine the effects of the acetone extract on cell migration, we used a wound healing assay. In brief, C-6 glioma cells were seeded into a culture dish and allowed to grow to 70% confluency. A wound was introduced into the monolayers using a sterile tip and washed to remove the cell debris. The cells were then allowed to grow in the culture media with or without the acetone extract. After 0, 12, and 24 h of treatment with acetone extract, the wound area was examined under the phase contrast microscope.

### 2.13. Assay for Reactive Oxygen Species Generation

Whether the acetone extract has the potential to generate reactive oxygen species (ROS) in the C-6 glioma cells was examined by flow cytometry [28]. Cells were treated with acetone extract (1–30 µg/mL) for 1 h. After the termination of the treatment time, cells were washed and stained with 10 µM 2′,7′-dichlorofluorescin diacetate (H_2_DCFDA) for 15 min in the dark. The stained cells were examined under fluorescence microscope and also by flow cytometry using FACScan. The analysis of flow cytometry data was carried out using Cell Quest software (Becton Dickinson, San Jose, CA, USA).

### 2.14. Gas Chromatography-Mass Spectrometry

Shimadzu Make GC-MS-TQ-8030 instrument (Tokyo, Japan) was used for the gas chromatography-mass spectrometry (GC-MS) analyses of different fractions of *Curcuma raktaknda* (hexane extract, ethyl acetate extract, and acetone extract). The compounds were separated using nonpolar Rxi 5 Sil MS capillary column in full scan mode with injector mode-splitless and quadra pole mass selective detector (MSD). The injection temperature was set at 250 °C while GC-MS interface temperature at 250 °C. An aliquot of 1 μL volume was injected into the column. Helium was used as a carrier gas (pressure: 57.5 KPa, flow rate: 1 mL/min). Mass spectra were detected at 70 eV. The temperature programming was set as follows: column temperature was started from 60 °C (held for 2 min) and linearly increased by 5 °C/min to 200 °C (held for 2 min); after that it was increased by 3 °C/min to 220 °C (held for 1 min); further it was increased by 6 °C/min to 250 °C (held for 7 min). Total GC running time was 51.67 min. The compounds were identified by comparing mass spectra of each peak with NIST5 and WILEY libraries.

## 3. Results

The overall goal of this study was to examine the anti-cancer potential of the extract from *C. raktakanda* that has demonstrated larvicidal activities. Most of the experiments were performed using C-6 glioma cell lines. We also used breast (MDA-MB-231, MCF-7) and cervical (HeLa) cancer cell lines to determine the specificity of the extract. The underlying mechanism and the potential components from the extract were also examined. 

### 3.1. Curcuma raktakanda Suppresses Viability and Reduces Long-Term Colony Formation of Glioma Cells 

**Figure 1:**
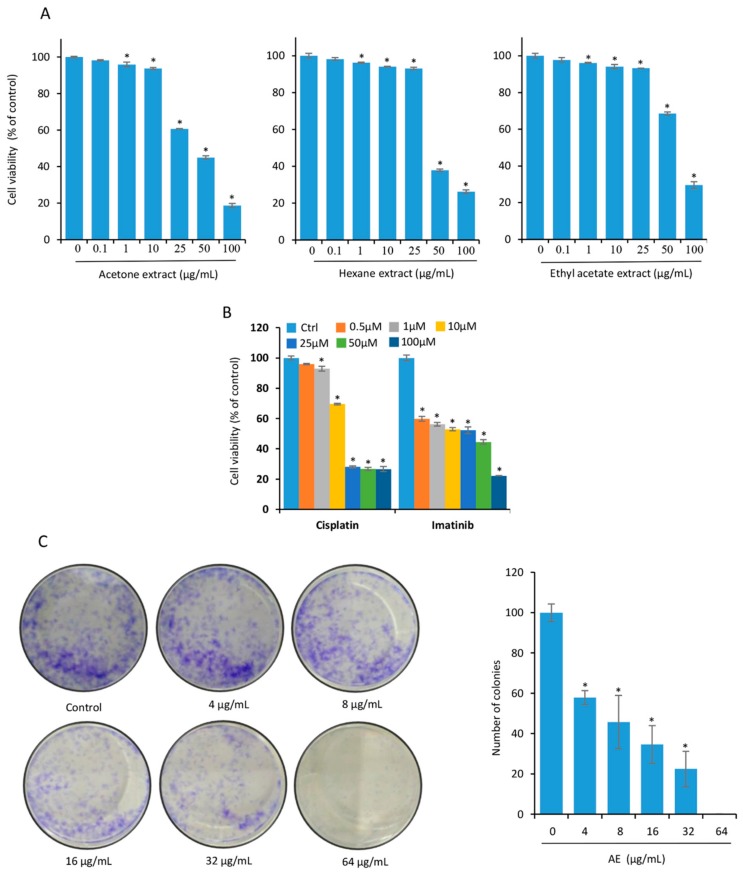
The rhizome extract of *C. raktakanda* suppresses viability and long-term colony formation of glioma cells. (**A**) C-6 glioma cells were exposed to different concentrations (µg/mL) of CR extracts (acetone, hexane, and ethyl acetate) for 48 h. The proliferation of cells was examined by 3-[4,5-dimethylthiazol-2-yl]-2,5-diphenyl tetrazolium bromide (MTT) assay. Note that the acetone extract was more potent as compared to hexane and ethyl acetate. (**B**) Cisplatin and imatinib were used as positive controls. (**C**) C-6 cells (1000 cells/well) were treated with indicated concentrations (µg/mL) of the acetone extract for 6 h. After seven days, the colonies were stained with 0.1% crystal violet and counted manually. A concentration dependent reduction in the number of colonies was observed after treatment with acetone extract. Where indicated, the values are mean ± SE from three experiments. *- indicates the significance of difference compared to the control group; *P* < 0.05. AE, acetone extract; CR, *Curcuma raktakanda*.

We used three different extracts (acetone, hexane, and ethylacetate) from the rhizomes of *C. raktakanda*. The cytotoxic potential of the extract was examined by measuring mitochondrial reductase activity using MTT as the substrate. We observed that the viability of C-6 glioma cells was suppressed by the three extracts in a concentration dependent manner (Figure 1A). However, the acetone extract was more effective when compared to the hexane and ethyl acetate extracts. For example, at a concentration of 25 µg/mL, 39% suppression in the viability was observed by the acetone extract. However, 7% suppression in the viability was observed by both hexane and ethyl acetate extracts. Furthermore, the half maximal inhibition (IC-50) values after treatment of C-6 cells for 48 h was found to be 32.97 µg/mL (acetone extract), 40.63 µg/mL (hexane extract), and 51.65 µg/mL (ethyl acetate extract). Therefore, we used the acetone extract (AE) for most experiments. Under similar experimental conditions, cisplatin and imatinib (positive controls) suppressed the viability of C-6 cells in a dose dependent manner (Figure 1B). 

Next, we examined the colony forming ability of C-6 glioma cells without and with AE treatment. Cells were exposed to 4, 8, 16, 32, and 64 µg/mL AE for 6 h. Cells were then washed and allowed to form colonies for 7 days. The extract significantly suppressed the number of colonies at a concentration as low as 16 µg/mL (Figure 1C). At 64 µg/mL, almost no colonies were observed. 

### 3.2. Acetone Extract induces Apoptosis in Glioma Cells

**Figure 2:**
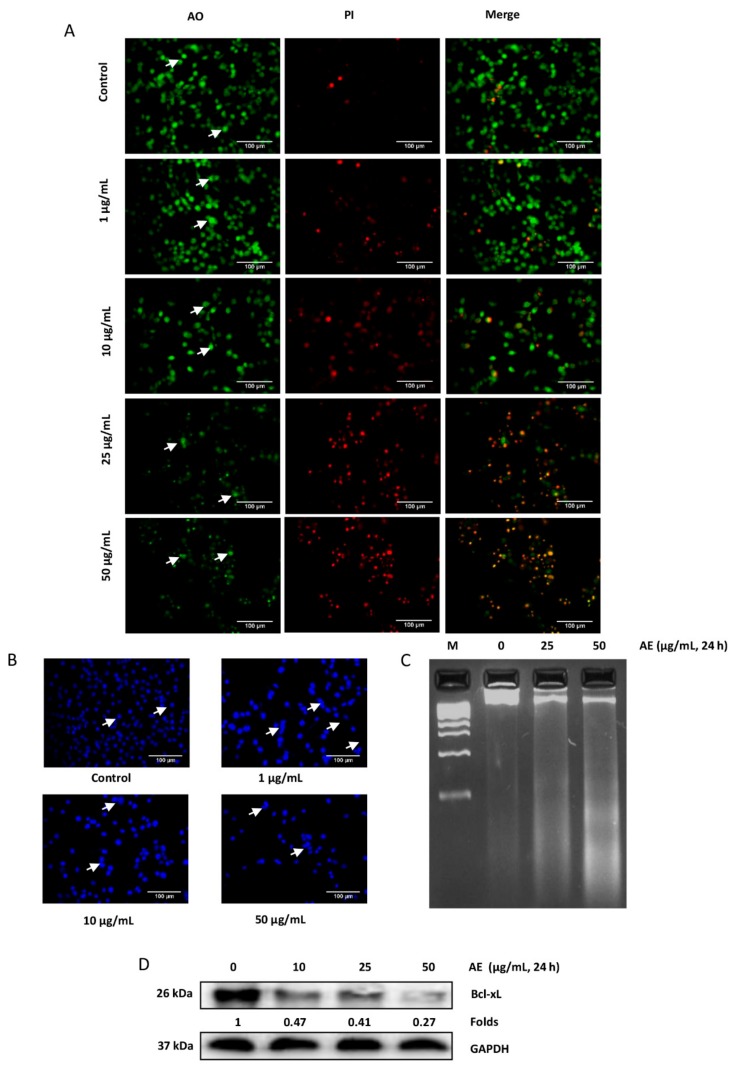
Acetone extract induces apoptosis in glioma cells. (**A**) C-6 glioma cells were exposed to different concentrations (µg/mL) of acetone extract. After 24 h, cells were stained with acridine orange (AO)/propidium iodide (PI) (10 µg/mL) and observed under fluorescence microscope. As indicated by arrows, control cells exhibited round to oval shaped green nucleus. In treated cells, orange to red fluorescence was observed with condensed and fragmented nucleus. (**B**) C-6 cells were treated with indicated concentration (µg/mL) of acetone extract. After 24 h, cells were fixed, permeabilized and mounted with DAPI. While fragmentation was observed in the treated cells, round and oval shaped nuclei were observed in the control cells as shown by arrows. (**C**) C-6 cells were exposed to acetone extract for 24 h, DNA was isolated and electrophoresed on agarose gel. Note the presence of DNA smear in the treated cells at higher concentration. (**D**) C-6 cells were exposed to indicated concentrations (µg/mL) of acetone extract for 24 h and the expression of Bcl-xL was examined in the whole cell extract by western blotting. Glyceraldehyde-3-phosphate dehydrogenase (GAPDH) was used as an internal control. Values below the blot indicate fold change in the Bcl-xL expression compared to control. The scale bar represents 100 µm. AE, acetone extract.

Apoptosis, a process of programmed cell death, is a normal physiological process. However, cancer cells have developed mechanisms to evade apoptosis. The induction of apoptosis selectively in cancer cells is one of the potential strategies of cancer therapy. Numerous assays were performed to examine the apoptosis inducing potential of AE. First, we examined the changes in the morphology of the cells by AO/PI dual staining. While AO can enter both live and dead cells, PI enters only dead cells with compromised mitochondria. Acridine orange stains all nucleated cells to generate green fluorescence. Propidium iodide stains dead nucleated cells to generate red fluorescence. Thus, the live nucleated cells produce green fluorescence, while dead nucleated cells produce red fluorescence. An increase in the concentration of AE was associated with a significant decrease in the number of viable cells. At a concentration as low as 10 µg/mL, features of early apoptosis such as membrane blebbing and nuclear condensation were observed after 24 h of treatment (Figure 2A). To confirm the observations obtained by AO/PI staining, we stained the control and treated cells with DAPI. A normal, oval, and round shaped nuclei was observed in control cells (Figure 2B). However, AE treated cells exhibited nuclear fragmentation and chromatin condensation (Figure 2B). One hallmark of late apoptosis is the cleavage of genomic DNA into oligonucleosomal fragments. Degraded DNA can be visualized by agarose gel electrophoresis. While the DNA from control cells was intact, DNA smear was observed from AE treated cells (Figure 2C). Cancer cells, including glioma, have evolved mechanism to survive. The suppression of Bcl-xL expression is one potential strategy to induce apoptosis in cancer cells. Whether AE can modulate Bcl-xL expression in C-6 glioma cells was examined by western blot analysis. While Bcl-xL was abundant in control cells, AE induced a concentration dependent decrease in the expression of Bcl-xL after 24 h of treatment (Figure 2D). Overall, these results suggest the apoptosis inducing potential of AE in C-6 glioma cells. 

### 3.3. Acetone Extract Induces Cell Cycle Arrest and Lowers Mitochondrial Membrane Potential in Glioma Cells 

**Figure 3:**
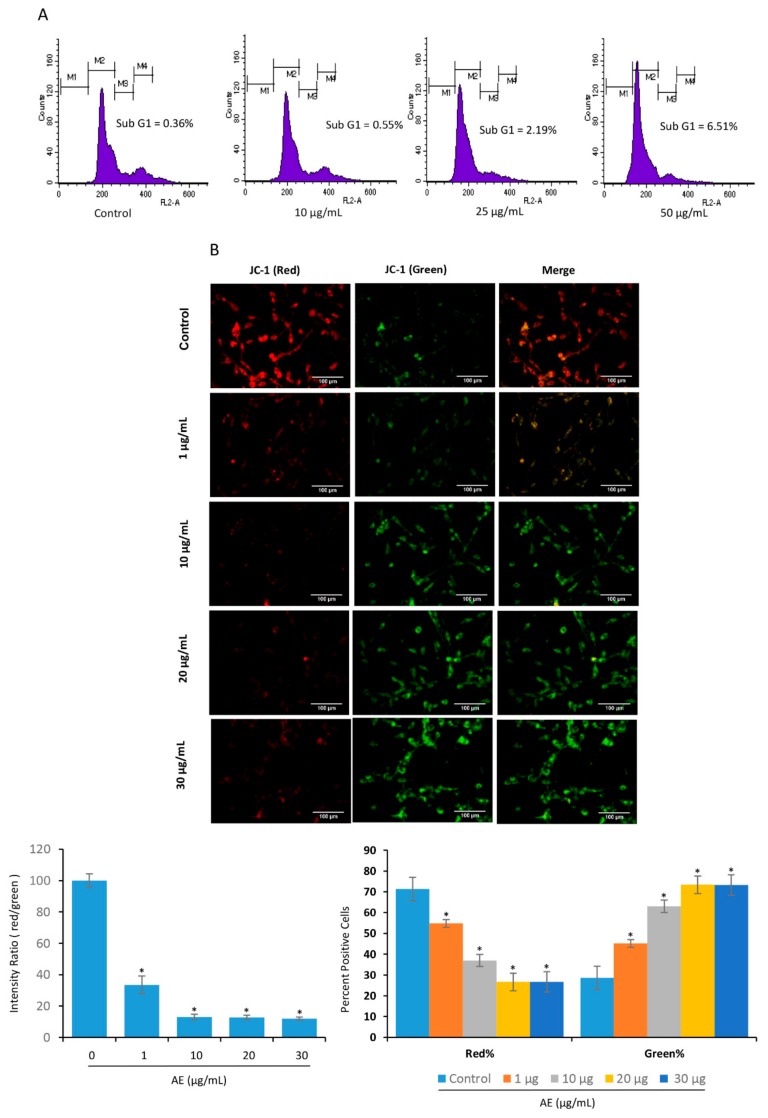
Acetone extract induces cell cycle arrest and lowers mitochondrial membrane potential in glioma cells. (**A**) C-6 glioma cells were exposed to indicated concentrations (µg/mL) of acetone extract for 24 h. Cells were then fixed and washed with PBS before staining with PI. The population at different phases of cell cycle were examined by flow cytometry. Note an increase in the sub-G1 population at the higher doses of acetone extract. (**B**) C6 cells were treated with the indicated concentrations (µg/mL) of the acetone extract for 24 h and then stained with JC-1 (10 µg/mL). The cells were then washed and observed under fluorescence microscope. In control cells, the intense red fluorescence indicates high mitochondrial membrane potential. The presence of green fluorescence in treated cells suggests depolarized mitochondria. A concentration dependent decrease in the ratio of red/green fluorescence was observed (lower left). While red stained cells decreased in a concentration dependent manner, a significant increase in the green stained cells was observed (lower right). Where indicated, the values are mean ± SE from three experiments. *- indicates the significance of difference compared to the control group; *P* < 0.05. The scale bar represents 100 µm. AE, acetone extract.

Next, we examined the sub-G1 population in the cells treated with or without AE. The treatment of C-6 cells with AE was associated with a modest increase in sub-G1 population (Figure 3A). One hallmark of early apoptosis is an irreversible reduction in mitochondrial membrane potential (MMP). At high MMP, JC-1 dye aggregates and produces red to orange colored fluorescence. JC-1 dye exists in monomeric form and produces green fluorescence at low MMP. In control glioma cells, red fluorescence was observed (Figure 3B, upper). In treated cells, an increase in green fluorescence was observed by increasing the concentration of AE (Figure 3B, lower). However, the red stained cells were decreased with an increase of AE concentration (Figure 3B, lower). Similarly, a concentration dependent decrease in the ratio of red/green intensity was observed with an increase of AE concentration. These observations suggest that the mitochondria are involved in the induction of apoptosis by AE in C-6 glioma cells.

### 3.4. Acetone Extract Suppresses the Migration of Glioma Cells 

**Figure 4:**
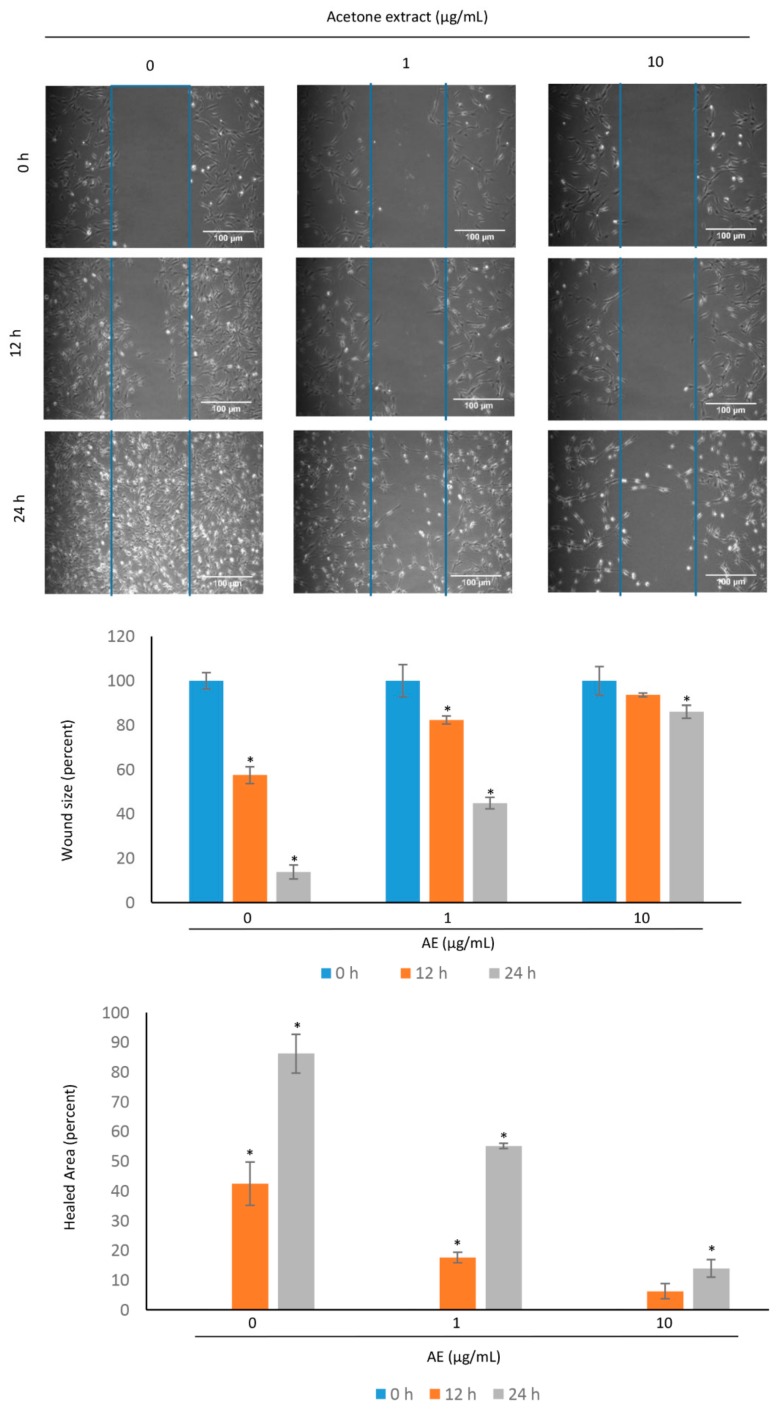
Acetone extract suppresses the migration of C-6 glioma cells. The sterile microtips were used to scratch over the cells with 70% confluency. The different concentrations (µg/mL) of acetone extract was then provided to the cells. The scratched area was examined under phase contrast microscope. The percentage of wound size and healed area was measured at different time intervals. The values are mean ± SE from three experiments. *- indicates the significance of difference compared to the control group; *P* < 0.05. Note a decrease in the migration potential of C-6 cells after treatment with acetone extract. The scale bar represents 100 µm. AE, acetone extract.

The glioma is characterized by local invasion. Before invasion, cells are detached from the primary tumor and migrate to the surrounding normal brain tissue. C-6 cells were exposed to different concentrations of AE (1, 10 µg/mL) and the migration of the cells was examined in the normal and treated cells after 12 h and 24 h (Figure 4). In control cells, the wound area was occupied by migrated cells with an increase of time. However, less cells were migrated to the wound area in the group treated with AE extract. Similarly, there was a significant increase in the healed area over time in the control cells. The increase in the healed area in the treated cells was less as compared to that observed in the control cells. These results suggest that the AE suppresses the migration of glioma cells. However, the migration of C-6 glioma cells were minimally affected by 1 µg/mL AE. 

### 3.5. Acetone Extract Induces Reactive Oxygen Species Generation in Glioma Cells

**Figure 5:**
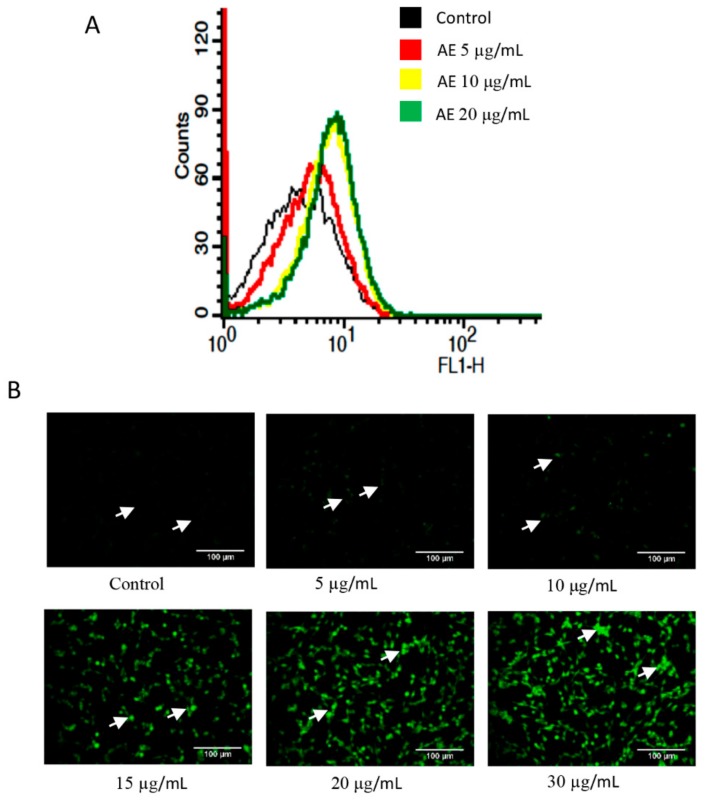
Acetone extract induces reactive oxygen species (ROS) generation. (**A**) C-6 cells were treated with indicated concentrations (µg/mL) of acetone extract for 1 h. The cells were then stained with H_2_DCFDA (10 µM) for 30 min and ROS generation was measured by flow cytometry. (**B**) The stained cells were also visualized under fluorescence microscope. The arrows indicate DCFDA stained cells in the control and treated groups. Note a concentration dependent increase in the level of ROS (DCFDA stained cells) after treatment with acetone extract. The scale bar represents 100 µm. AE, acetone extract.

Cancer cells have the inherent property of increased ROS generation that can be exploited to induce ROS selectively. Next, we examined the potential of AE on ROS generation in C-6 glioma cells. The cells were exposed to different concentrations of AE for 1 h and stained with H2DCFDA. The ROS generation was measured by flow cytometry and also by fluorescence microscopy. The ROS generation in control and in 5 µg/mL AE treated cells was minimal (Figure 5). However, an increase in ROS generation was observed with an increase in AE concentration. For example, a respective 1.6-fold and 1.7-fold increase in ROS generation was observed at 10 µg/mL and 20 µg/mL AE, respectively. 

### 3.6. Curcuma raktakanda Reduces the Viability of Breast and Cervical Cancer Cells

**Figure 6:**
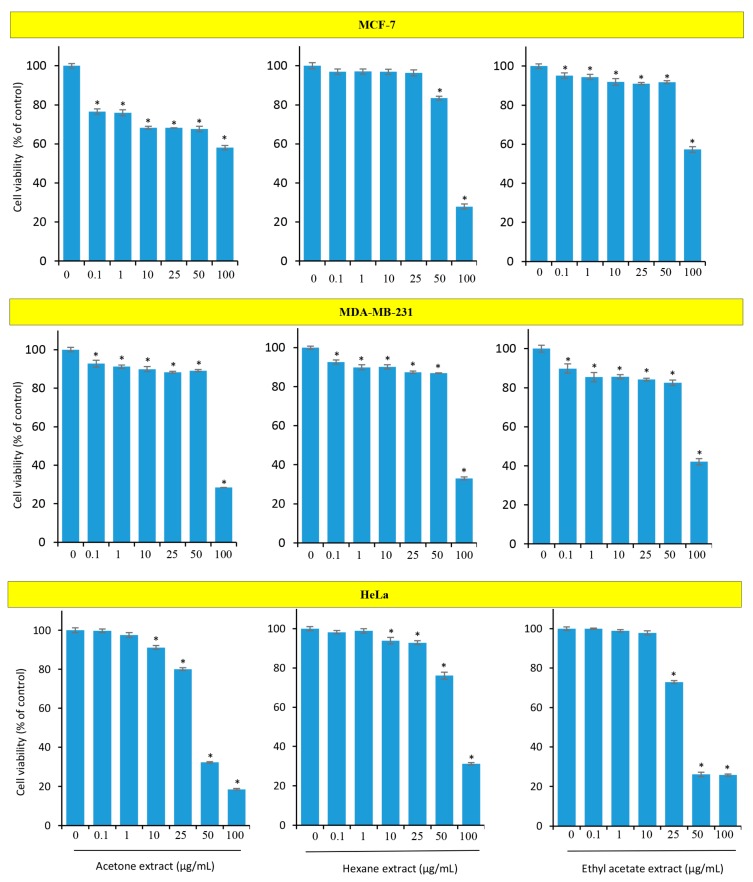
The rhizome extract of *C. raktakanda* suppresses the proliferation of cancer cells from diverse origin. Breast (MDA-MB-231, MCF-7) and cervical (HeLa) cells were exposed to different concentrations (µg/mL) of CR extracts (acetone, hexane, ethyl acetate) for 48 h. The viability of cells was examined by mitochondrial membrane potential (MTT) assay. Note that the cancer cells exhibited varied degree of sensitivity to the three extracts. Values are mean ± SE from three experiments. *- indicates the significance of difference compared to the control group; *P* < 0.05.

Next, we examined the cytotoxic activities of *C. raktakanda* in cancer cells other than glioma. For this, we used breast (MDA-MB-231, MCF-7) and cervical (HeLa) cancer cell lines. Cells were exposed to different extracts of *C. raktakanda* and proliferation was examined by MTT assay. The three extracts suppressed the proliferation of cancer cells in a concentration dependent manner (Figure 6). However, the cancer cells exhibited varied degrees of sensitivity to the extracts. For example, at lower concentrations (0.1–50 µg/mL), acetone extract was more effective compared to hexane and ethyl acetate extracts in MCF-7 cells. However, at the highest tested concentration (100 µg/mL) of the hexane extract was more effective. In MDA-MB-231 cells, the ethyl acetate extract was relatively more effective compared to the acetone and hexane extracts. In HeLa cells, acetone extract was more effective compared to the other two extracts. Overall, these results suggest that the cancer cells differ in their sensitivity to the *C. raktakanda*.

### 3.7. Gas Chromatography-Mass Spectrometry Analysis of Curcuma raktakanda Rhizome Fractions

A previous study demonstrated the larvicidal activity of *C. raktakanda* [20]. However, the phytoconstituents of this plant have not been analyzed. Finally, we analyzed the phytoconstituents of three extracts by GC-MS. The identification and interpretation of GC-MS data was accomplished using the data from National Institute Standard and Technology (Gaithersburg, MD, USA). A respective 26, 21, and 30 peaks were obtained from acetone, hexane, and the ethyl acetate fraction, respectively (Table 1, Table 2 and Table 3). The major compounds in hexane fractions were: beta-Elemenone (16.84%), Cycloprop[e]indene-1a,2(1H)-dicarboxaldehyde,3a,4,5,6,6a,6b-hexahydro-5,5,6b-trimethyl (1a.alpha.,3a.beta.,6a.beta.,6b.alpha) (10.49%), Alloaromadendreneoxide-(1) (8.47%), and 5,8-dihydroxy-4a-methyl-4,4a,4b,5,6,7,8,8a,9,10-decahydro-2(3*H*)-phenanthrenone (8.33%). The ethyl acetate fraction contained mainly 2-phosphabicyclo[3.1.0]hex-3-ene, 6,6-dimethyl-2,3,4-triphenyl-, (endo)- (16%), desmethylnomifensine (12.6%), 1,1,6-trimethyl-3-methylene-2-(3,6,9,13-tetramethyl-6-ethenye-10,14-dimethylene-pentadec-4-enyl) cyclohexane (9.48%), and cobalt, hexamethylbenzene-pentamethylcyclopentadienyl (8.67%). The major components in the acetone fraction were reported to be tetracontane (38.53%), (-)-(1*R*,2*R*,4a*S*,8a*S*)-1-(2-hydroxyethyl)-2,5,5,8a-tetramethyldecahydro-2-naphthalenol (9.10%) and dotriacontane (6.16%). 

## 4. Discussion

Although much has been published on the golden spice, turmeric (*C. longa*), very little is known about other Curcuma species. Curcumin, which constitutes 2–5% of turmeric, is one of the most active components [18]. Both curcumin and turmeric are known to modulate a number of cancer related targets by preclinical and clinical studies [16,29]. The genus Curcuma constitutes more than 130 species. Whether *C. raktakanda* exhibit anti-cancer activities is unknown. Therefore, the focus of the current study was to examine the biological activities of *C. raktakanda* against glioblastoma cells.

We observed that the three extracts of *C. raktakanda* exhibited activities against C-6 glioblastoma cell lines. However, the acetone extract was more effective compared to the other two. Further, the acetone extract induced apoptosis and suppressed proliferation of C-6 glioma cells. For the first time, we found that the acetone extract inhibited the expression of cell survival protein Bcl-xL. Glioblastoma cells are known to express high levels of Bcl-xL [30]. The Bcl-xL promotes tumorigenesis partly through the nuclear factor-kappa B/chemokine (C-X-C motif) ligand 8 (NF-κB/CXCL8) mediated angiogenesis [31,32]. Additionally, the survival of several cancer types primarily depends on the Bcl-xL expression [33,34]. The observed inhibitory effects of the extract on tumor cell survival could be due to the suppression of the Bcl-xL expression. The suppression of Bcl-xL expression by the acetone extract may be due to its inhibitory effects on NF-κB activation. The presence of membrane blebbing, nuclear condensation, and accumulation of cells at the sub-G1 phase after treatment with *Curcuma raktakanda* (CR) extract further supports its negative effects on the survival of C-6 glioblastoma cells.

The main cause of glioblastoma mortality is not the primary tumor but because the tumor invades locally. This leads to the ineffectiveness of current treatment options and recurrence of the tumor. The invasion involves detaching the cells from the primary tumor followed by migration in the surrounding normal brain tissue. In our observations, CR extract was found to suppress the migration of C-6 glioma cells. The process of migration and invasion involved the synthesis of proteases such as metalloproteinases that degrade extracellular-matrix (ECM) components selectively [35]. Whether CR extract modulates these proteases remains to be elucidated. 

The extract used in the current study is a mixture of several components. The GC-MS analyses indicated that the three extracts are different in terms of phytochemical constituents. The major component of acetone fraction such as tetracontane, dotriacontane, and hexatriacontane has not been reported to exhibit anti-cancer activities previously. However, minor components of the acetone fraction such as pentacosane and eicosane are reported to exhibit anti-cancer activities. In a previous study, the essential oil from the leaves of *Malus domestica* containing pentacosane exhibited activities against C-6, A549 (lung carcinoma), CHOK1 (ovarian), and THP-1 (acute monocytic leukemia) cells [36]. Similarly, eicosane (macrolide) produces cytotoxicity both in vitro and in nude mice bearing human ovarian carcinoma cells [37]. Eucalyptol and elemene, identified in the hexane and ethylacetate extracts, are also known to exhibit anti-cancer activities [38,39,40,41]. Whether the components of the extract interact in an additive, synergistic, or antagonistic manner remains to be explored.

We also observed that the CR extract inhibits the viability of not only glioblastoma cells but also breast cancer cells and cervical cancer cells. These observations suggest the broad-spectrum effects of the extract. We observed that AE induces ROS generation in C-6 glioblastoma cells. The role of ROS in the regulation of cancer associated cell signaling pathways is well established [42]. Further, chemotherapeutic agents and nutraceuticals mediate their effects through the production of ROS [43,44]. Implication of all these suggest that ROS act as a double-edged sword during cancer pathogenesis [45]. Whether ROS is required for the anti-cancer activities of the acetone extract remains to be elucidated. The ability of CR extract to disrupt mitochondrial membrane potential suggest that the apoptosis is mediated through the mitochondria.

A previous study demonstrated that the CR extract exhibit larvicidal activities [20]. Thus, our observations provide a possibility that the non-cancer agents such as larvicides could be repurposed for anti-cancer activities. The application of larvicides in cancer therapy may appear unconventional. However, drug repurposing is an emerging novel approach to significantly reduce the time involved in drug development process [46]. Similar to our observation, deltamethrin, deguelin, destruxin, and rotenone type of insecticides have also demonstrated potential against cancer cells [47]. Similarly, rapamycin which was originally developed as a macrolide antibiotic exhibits anti-cancer activities [48]. Minocycline is another antibiotic being tested for its activities against ovarian cancer, glioma, and numerous other cancer types [49]. 

The development of drug resistance is a major hurdle in the therapy of several cancer types including glioblastoma [50]. Our unpublished observations suggest that the acetone extract can sensitize C-6 cells to doxorubicin at higher concentrations. These observations provide an opportunity that the AE can be used in combination with existing drugs to overcome chemoresistance. Cancer related genes such as NF-κB and cell survival proteins are involved in the development of drug resistance [51]. A possibility that the suppression in Bcl-xL expression by AE sensitizes cancer cells to doxorubicin cannot be ruled out. Whether AE modulate NF-κB activation remains to be elucidated. 

In conclusion, our observations suggest that the rhizome extract from *C. raktakanda* exhibit anti-carcinogenic activities. The components from the extract responsible for all these activities remains to be elucidated. The generation of ROS by the extract may contribute to its anti-cancer activities. Our observations are important as very little is known about this plant. One limitation of the current study is that the actual component of the extract responsible for the observed anticancer effects is not known. However, with several components, the mixture can target multiple glioblastoma associated pathways. This may offer an advantage as the glioblastoma is caused by dysregulation of multiple genes and thus multi-targeting approach is required. We are currently working on the constituents and the underlying mechanism by which this plant exhibits broad-spectrum anti-cancer activities.

## Figures and Tables

**Table 1:** Gas chromatography-mass spectrometry (GC-MS) analysis of acetone extract of *Curcuma raktakanda*.

S. No.	Peak No.	Retention Time (min)	Area %	Compound Name	Molecular Weight	Molecular Formula
1	1	18.985	0.84	13,17-Dimethylhentriacontane	464.907	C_33_H_68_
2	2	19.331	0.71	Hexacosane	366.718	C_26_H_54_
3	3	20.129	0.75	Eicosane	282.5475	C_20_H_42_
4	4	20.541	2.41	Tetracontane	563.096	C_40_H_82_
5	5	21.219	1.17	Tetracontane	563.096	C_40_H_82_
6	6	21.646	0.80	Pentacosane	352.691	C_25_H_52_
7	7	22.129	0.12	Pentacosane	352.691	C_25_H_52_
8	8	22.494	0.26	Dotriacontane	450.88	C_32_H_66_
9	9	22.860	2.15	Tetracontane	563.079	C_40_H_82_
10	10	27.890	0.08	Acetic acid, 1-[2-(2,2,6-trimethyl-bicyclo[4.1.0]hept-1-yl)-ethyl]-vinyl ester	250.376	C_16_H_26_
11	11	39.280	1.02	Celidoniol, deoxy-	424.798	C_29_H_60_
12	12	40.494	0.20	Nonacosane	408.799	C_29_H_60_
13	13	40.727	0.20	Pentatriacontane	492.961	C_35_H_72_
14	14	41.535	0.54	Heneicosyl pentafluoropropionate	458.5890	C_24_H_43_F_5_O_2_
15	15	42.352	0.40	1,30-Triacontanediol	454.812	C_30_H_62_
16	16	43.000	4.14	(2e)-1-methyl-3-(2,6,6-trimethyl-2-cyclohexen-1-yl)-2-propenyl acetate	236.355	C_15_H_24_O_2_
17	17	43.100	6.16	Dotriacontane	450.88	C_32_H_66_
18	18	43.286	38.53	Tetracontane	563.096	C_40_H_82_
19	19	44.396	0.33	Tetratriacontyl heptafluorobutyrate	690.9421	C_38_H_69_F_7_O_2_
20	20	44.620	0.21	Pentatriacontane	492.961	C_35_H_72_
21	21	44.890	0.19	1-Hentetracontanol	593.122	C_41_H_84_O
22	22	45.290	0.28	Tetrapentacontane, 1,54-dibromo-	917.266	C_54_H_108_Br_2_
23	23	46.220	1.67	Hexatriacontane	506.988	C_36_H_74_
24	24	46.746	0.52	Hexatriacontane	506.988	C_36_H_74_
25	25	48.345	9.10	(-)-(1R,2R,4aS,8aS)-1-(2-Hydroxyethyl)-2,5,5,8a-tetramethyldecahydro-2-naphthalenol	308.499	C_20_H_36_
26	26	48.501	27.22	Tetracontane	563.096	C_40_H_82_

The compounds were detected using database NIST08s.LIB (Gaithersburg, MD, USA), WILEY8.LIB (Hoboken, NJ, USA), and PUBCHEM (NCBI, MD, USA).

**Table 2:** GC-MS analysis of hexane extract of *C. raktakanda*.

S. No.	Peak No.	Retention Time (min)	Area %	Compound Name	Molecular Weight	Molecular Formula
1	1	8.277	4.4	Eucalyptol	154.249	C_10_H_18_O
2	2	11.516	5.17	(+)-2-Bornanone	152.2334	C_10_H_16_O
3	3	16.746	0.88	.delta.-Elemene	204.357	C_15_H_24_
4	4	18.206	7.44	2,4-diisopropenyl-1-methyl-1-vinylcyclohexane	204.351	C_15_H_24_
5	5	19.222	5.14	.gamma.-Elemene	204.3511	C_15_H_24_
6	6	20.739	2.45	beta.-Selinene	204.357	C_15_H_24_
7	7	20.858	8.2	Benzofuran, 6-ethenyl-4,5,6,7-tetrahydro-3,6-dimethyl-5-isopropenyl-, trans-	216.3187	C_15_H_20_O
8	8	23.398	16.84	beta.-Elemenone	218.34	C_15_H_22_O
9	9	24.044	2.18	1H-Cycloprop[e]azulen-7-ol, decahydro-1,1,7-trimethyl-4-methylene-, [1ar-(1a.alpha.,4a.alpha.,7.beta.,7a.beta.,7b.alpha.)]-	220.3505	C_15_H_24_O
10	10	25.539	3.91	3,7-Dimethyl-10-(1-methylethylidene)-3,7-cyclodecadien-1-one	218.3346	C_15_H_22_O
11	11	26.899	8.33	5,8-Dihydroxy-4a-methyl-4,4a,4b,5,6,7,8,8a,9,10-decahydro-2(3H)-phenanthrenone	250.333	C_15_H_22_
12	12	27.652	10.49	Cycloprop[e]indene-1a,2(1H)-dicarboxaldehyde, 3a,4,5,6,6a,6b-hexahydro-5,5,6b-trimethyl-, (1a.alpha.,3a.beta.,6a.beta.,6b.alpha	232.323	C_15_H_20_O_2_
13	13	28.643	2.17	Elemene	204.3511	C_15_H_24_
14	14	28.958	6.54	Acetic acid, 6-(1-hydroxymethyl-vinyl)-4,8a-dimethyl-3-oxo-1,2,3,5,6,7,8,8a-octahydronaphthalen-2-yl ester	292.375	C_17_H_24_O_4_
15	15	29.872	0.72	Bufa-20,22-dienolide, 14,15-epoxy-3,5,16-trihydroxy-, (3.beta.,5.beta.,15.beta.,16.beta.)-	500.588	C_28_H_36_O_8_
16	16	30.033	1.07	Cyclohexane, 1-ethenyl-1-methyl-2-(1-methylethenyl)-4-(1-methylethylidene)-	204.3511	C_15_H_24_
17	17	31.13	1.41	n-Hexadecanoic acid	256.4241	C_16_H_32_O_2_
18	18	31.617	0.51	Hydroxydehydrostevic acid	318.457	C_20_H_30_O_3_
19	19	35.362	1.67	4,8,13-Cyclotetradecatriene-1,3-diol, 1,5,9-trimethyl-12-(1-methylethyl)-	306.49	C_20_H_34_O_2_
20	18	41.197	8.47	Alloaromadendrene oxide-(1)	220.3505	C_15_H_24_O
21	19	44.861	2.01	Longifolenaldehyde	220.356	C_15_H_24_O

The compounds were detected using database NIST08s.LIB (Gaithersburg, MD, USA), WILEY8.LIB (Hoboken, NJ, USA), and PUBCHEM (NCBI, MD, USA).

**Table 3:** GC-MS analysis of ethyl acetate extract of *C. raktakanda*.

S. No.	Peak No.	Retention Time (min)	Area %	Compound Name	Molecular Weight	Molecular Formula
1	1.	11.553	0.74	Bicyclo[2.2.1]heptan-2-one, 1,7,7-trimethyl-	152.2334	C_10_H_16_O
2	2.	12.262	0.28	Bicyclo[2.2.1]heptan-2-ol, 1,7,7-trimethyl-, (1S-endo)-	196.2860	C_12_H_20_O_2_
3	3.	18.197	1.14	2,4-Diisopropenyl-1-methyl-1-vinylcyclohexane	204.351	C_15_H_24_
4	4.	18.435	0.52	Hexadecane	226.41	C_16_H_34_
5	5.	19.219	0.68	.gamma.-Elemene	204.357	C_15_H_24_
6	6.	20.743	0.33	.beta.-Selinene	204.357	C_15_H_24_
7	7.	20.832	0.34	5-Isopropenyl-3,6-dimethyl-6-vinyl-4,5,6,7-tetrahydro-1-benzofuran	216.3187	C_15_H_20_O
8	8.	20.951	1.19	Heptadecane	240.4677	C_17_H_36_
9	9.	23.346	3.81	beta.-Elemenone	218.34	C_15_H_22_O
10	10.	24.041	0.61	Isospathulenol	220.356	C_15_H_24_O
11	11.	25.531	0.64	3,7-Dimethyl-10-(1-methylethylidene)-3,7-cyclodecadien-1-one	218.3346	C_15_H_22_O
12	12.	27.585	0.86	Cycloprop[e]indene-1a,2(1H)-dicarboxaldehyde, 3a,4,5,6,6a,6b-hexahydro-5,5,6b-trimethyl-, (1a.alpha.,3a.beta.,6a.beta.,6b.alpha	232.323	C_15_H_20_O_2_
13	13.	27.745	1.68	Norethindrone	298.426	C_20_H_26_O_2_
14	14.	27.864	1.25	(Albicanol) Decahydro-2-methylene-5,5,8a-trimethyl-1-naphthalenemethanol	222.366	C_15_H_26_
15	15.	28.63	0.88	9-t-Butyltricyclo[4.2.1.1(2,5)]decane-9,10-diol	224.339	C_14_H_24_
16	16.	30.031	0.7	gamma.-Elemene	204.357	C_15_H_24_
17	17.	30.387	0.81	Longifolenaldehyde	220.3505	C_15_H_24_O
18	18.	31.097	1.46	l-(+)-Ascorbic acid 2,6-dihexadecanoate	652.954	C_38_H_68_O_8_
19	19.	33.24	8.32	2-Hydroxy-4-isopropyl-7-methoxytropone	194.23	C_11_H_14_O_3_
20	20.	34.684	8.34	Pregn-4,16-diene-3,20-dione dimethoxime	314.469	C_21_H_30_O_2_
21	21.	35.367	1.24	4,8,13-Cyclotetradecatriene-1,3-diol, 1,5,9-trimethyl-12-(1-methylethyl)-	306.49	C_20_H_34_O_2_
22	22.	36.495	1.9	2H-Cyclohepta[b]furan-2-one, 6-[1-(acetyloxy)-3-oxobutyl]-3,3a,4,7,8,8a-hexahydro-7-methyl-3-methylene-	306.146	C_17_H_22_O_5_
23	23	38.001	2.03	4,7-Methanofuro[3,2-c]oxacycloundecin-6(4H)-one, 7,8,9,12-tetrahydro-3,11-dimethyl-	246.306	C_15_H_18_O_3_
24	24	39.499	0.9	Behenic alcohol	326.6000	C_22_H_46_O
25	25	41.176	9.48	1,1,6-trimethyl-3-methylene-2-(3,6,9,13-tetramethyl-6-ethenye-10,14-dimethylene-pentadec-4-enyl)cyclohexane	220.350	C_15_H_24_
26	26	42.513	16	2-Phosphabicyclo[3.1.0]hex-3-ene, 6,6-dimethyl-2,3,4-triphenyl-, (endo)-	136.194	C_8_H_12_N
27	27	43.429	8.67	Cobalt, hexamethylbenzene-pentamethylcyclopentadienyl-	329.39	C_20_H_30_Co
28	28	44.587	12.6	Desmethylnomifensine	224.307	C_15_H_16_N_2_
29	29	44.871	8.26	Longifolenaldehyde	220.3505	C_15_H_24_O
30	30	46.344	4.34	Furan, 2,5-bis(3,4-dimethoxyphenyl)tetrahydro-3,4-dimethyl-, [2S-(2.alpha.,3.beta.,4.alpha.,5.beta.)]-	372.461	C_22_H_28_O_5_

The compounds were detected using database NIST08s.LIB (Gaithersburg, MD, USA), WILEY8.LIB (Hoboken, NJ, USA), and PUBCHEM (NCBI, MD, USA)
